# Carbocysteine Modifies Circulating miR-21, IL-8, sRAGE, and fAGEs Levels in Mild Acute Exacerbated COPD Patients: A Pilot Study

**DOI:** 10.3390/ph15020218

**Published:** 2022-02-11

**Authors:** Maria Ferraro, Serena Di Vincenzo, Claudia Sangiorgi, Stefania Leto Barone, Sebastiano Gangemi, Luigi Lanata, Elisabetta Pace

**Affiliations:** 1 Institute for Biomedical Research and Innovation (IRIB)—Consiglio Nazionale delle Ricerche, 90146 Palermo, Italy; maria.ferraro@cnr.it (M.F.); serena.divincenzo@cnr.it (S.D.V.); san.85@hotmail.it (C.S.); 2 Institute of Translational Pharmacology (IFT)—National Research Council, 90146 Palermo, Italy; 3 Casa di Cura Orestano, 90138 Palermo, Italy; msletobarone@gmail.com; 4 Department of Clinical and Experimental Medicine, School and Operative Unit of Allergy and Clinical Immunology, University of Messina, 98125 Messina, Italy; gangemis@unime.it; 5 Dompè Medical Affair, 20122 Milan, Italy; Luigi.Lanata@dompe.com

**Keywords:** respiratory pharmacology, inflammation, antioxidants, COPD, exacerbations, carbocysteine

## Abstract

Patients with Chronic Obstructive Pulmonary Disease (COPD) periodically experience acute exacerbation (AECOPD). Carbocysteine represents a valid add on therapy in COPD by exerting antioxidant and anti-inflammatory activities. The in vivo effects of carbocysteine on inflammatory markers are not yet fully understood. The aims of this study were to assess: (i) miR-21, IL-8, soluble Receptor for Advanced Glycation End Products (sRAGE), and fluorescent Advanced Glycation End Products (fAGEs) in control subjects (*n* = 9), stable (*n* = 9), and AECOPD patients (*n* = 24); and (ii) whether carbocysteine modifies these markers and the functional parameters in mild AECOPD patients. Mild AECOPD patients received or not carbocysteine along with background inhalation therapy for 20 days. At the onset and at the end of the observation period, the following parameters were evaluated: FEV1, FEF25–75%, CAT questionnaire; miR-21 by Real Time PCR; IL-8 and sRAGE by ELISA; and fAGEs by spectro-fluorescence method. COPD patients showed higher levels of miR-21, IL-8, fAGEs and lower levels of sRAGE compared to that of controls. miR-21 inversely correlated with FEV1. IL-8 and fAGEs were significantly different in stable and exacerbated COPD patients. Carbocysteine improved symptoms, FEV1 and FEF25–75%, increased sRAGE, and reduced miR-21, IL-8, and fAGEs in mild AECOPD patients. The present study provides compelling evidence that carbocysteine may help to manage mild AECOPD by downregulating some parameters of systemic inflammation.

## 1. Introduction

Chronic Obstructive Pulmonary Disease (COPD) is characterized by chronic inflammation that leads to airflow limitation and remodeling of the airways. Continuous exposure to risk factors such as cigarette smoke represents a major risk factor. Oxidative stress plays an important role in the pathogenesis of COPD, and oxidative stress markers are increased in exhaled breath condensate, sputum, and blood of patients with COPD [1]. COPD patients periodically experience acute exacerbations (AECOPD), periods of acute worsening of respiratory symptoms due to inflammatory processes generally triggered by viral or bacterial infections. Frequent exacerbations are associated with a more rapid decline in lung function, a poorer quality of life, and an increase in mortality. For this reason, AECOPD have strong clinical and economic consequences, and it is important to find treatments that reduce their frequency and duration [2]. COPD exacerbations are defined according to Anthonisen criteria [3]. An exacerbation is defined as worsening of COPD symptoms for at least two consecutive days. It was classified as: mild, when patients do not require treatment with systemic corticosteroids and/or antibiotics; moderate, when treatment with systemic corticosteroids and/or antibiotics are required; and severe, when hospitalization or visit to the emergency care unit is required [4].

Several clinical studies demonstrated that the use of mucolytic agents associated with traditional therapy could reduce the frequency of exacerbations, and they represents a cost-effective therapy in patients who exacerbate frequently and produce sputum chronically [5]. Carbocysteine, one of the used mucolytic drugs, has anti-inflammatory and antioxidant actions [6,7]. However, there is a limited number of studies that analyze the efficacy of carbocysteine in specific phenotypes of patients or study the molecular mechanisms underlying its efficacy.

Several studies recognize the role of microRNAs (miRNAs) in the development and progression of chronic lung diseases [8]. miRNAs are small, noncoding RNAs that control gene expression through base-pairing with target mRNAs inducing degradation of mRNA or blocking its translation and the synthesis of the target protein. miRNAs are involved in a variety of cellular and molecular pathways associated with the initiation and progression of several diseases. Growing evidence indicates that circulating miRNAs are noninvasive molecular markers and predictors of disease activity [8]. MiR-21 is involved in different types of tumors [9,10] and diseases related to increased oxidative stress and inflammation [11,12]. COPD is a systemic inflammatory syndrome [13] and systemic miR-21 and IL-8 are both increased in COPD, acting as indicators of COPD severity [14]. Previous studies demonstrated that cigarette smoke extracts (CSE) increase oxidative stress, leading to miR-21 upregulation and consequent activation of ERK pathway and IL-8 expression in human bronchial epithelial cells [15]. IL-8 is elevated in the airways of COPD patients and has a central role in the COPD pathogenesis and symptoms [16]. This is because IL-8 promotes the recruitment and activation of neutrophils contributing to their infiltration and the consequent airway tissue damage.

Oxidative stress also leads to the formation of advanced glycation end products (AGEs), a class of covalently modified proteins, due to oxidation reactions involving sugars or their degradation products [17]. Fluorescent AGEs (fAGEs) are considered a representative subset of the entire AGE family [18]. The main biological effect resulting from the formation of these products is the loss of functionality of the modified proteins. AGEs activate the immune response; in fact, they are involved in the pathogenesis of autoimmune diseases [17]. Furthermore, AGEs can bind AGE receptor (RAGE), inducing the activation of its signaling pathway, which in turn promotes damaging and proinflammatory effects. RAGE is an important mediator in different lung diseases, such as asthma, pulmonary fibrosis, lung cancer, COPD, and acute lung injury [19]. It is a protein of the immunoglobulin superfamily and exists in two main forms: membrane-bound RAGE (mRAGE) and soluble RAGE (sRAGE). mRAGE is a transmembrane receptor and its interaction with AGEs activates a proinflammatory cascade through NF-κB [20]. sRAGE is a decoy receptor for AGEs with anti-inflammatory properties because it competes with mRAGE for the binding of ligands. sRAGE is decreased in the circulation of COPD patients compared to that of control groups and shows a positive association with lung function [21]. AECOPD patients show low levels of sRAGE compared to that of the period when they are clinically stable [22].

Several clinical studies evaluated the use of alternative therapies to reduce the frequency and severity of AECOPD, but the correlation between changes in miR-21, IL-8, sRAGE, and fAGEs in serum of mild AECOPD patients and the clinical efficacy of traditional therapy with an additional mucolytic drug were never evaluated. The aim of this study was to confirm whether patients with COPD (stable and exacerbated) show altered levels of miR-21, IL-8, sRAGE, and fAGEs in serum. Furthermore, the study aimed at investigating whether administration of carbocysteine at short-term cycle, as an additional drug to the background inhalation therapy, in a more selected group of COPD patients with frequent exacerbation and mucus hypersecretion, may improve lung function and symptoms during an acute mild COPD exacerbation together with a modulation of miR-21, IL-8, sRAGE, and fAGEs. In addition, the study was performed in COPD patients with a mild exacerbation to better discriminate the effect of carbocysteine, since these patients do not require treatment with systemic corticosteroids and/or antibiotics.

## 2. Results

### 2.1. Stable and Exacerbated COPD Show Increased Levels of Circulating miR-21, IL-8 and fAGEs and Lower Levels of sRAGE Compared to Healthy Controls

**Figure 1:**
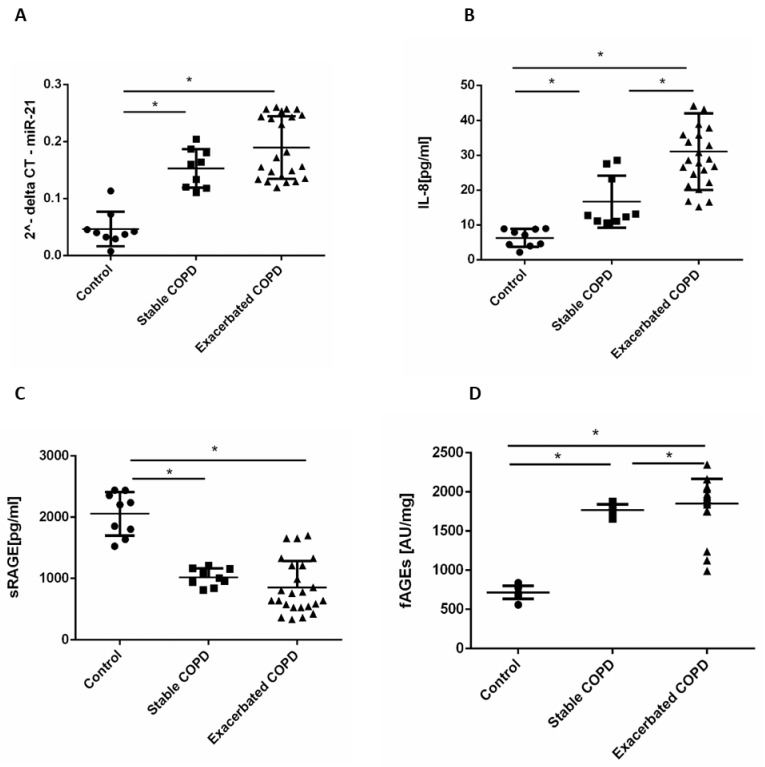
MiR-21, IL-8, soluble Advanced Glycation End Products (sRAGE), and fluorescent advanced glycation end products (fAGEs) expression in control, stable, and exacerbated COPD. Total RNA was purified from human serum, and miR-21 expression was assessed by real-time PCR. Results are expressed as 2^-delta CT (**A**). measurement of IL-8 and sRAGE in serum were determined by ELISA. Results are expressed as pg/mL (**B**,**C**). Measurement of fAGEs in serum was determined by fluorescence spectroscopy analysis. Results are expressed as Arbitrary Units (AU)/mg of total protein (**D**). Control *n* = 9, Stable COPD *n* = 9, Exacerbated COPD *n* = 24. * *p* < 0.05 Mann–Whitney test.

MiR-21 plays a central role in inflammation [12] and it is a good indicator of COPD severity [14]. The levels of circulating miR-21 in stable and exacerbated COPD patients were initially tested. As shown in Figure 1A, circulating miR-21 was higher in the serum of stable and exacerbated COPD patients than in controls. In particular, circulating miR-21 in stable COPD increased 3-fold compared to that of controls, while in exacerbated COPD it increased 4-fold compared to that of controls.

Then, the levels of IL-8 in serum from controls, stable, and exacerbated COPD patients were assessed. Our data showed that IL-8 levels were significantly increased in stable and exacerbated COPD patients compared to controls (Figure 1B). In addition, exacerbated COPD patients showed higher levels of IL-8 compared to that of stable COPD patients. In particular, the levels of IL-8 in stable COPD increased 2.65-fold compared to that of controls, in exacerbated COPD they increased 5-fold compared to that of controls, while in exacerbated COPD they increased 1.9-fold compared to that of stable COPD.

AECOPD patients show low levels of soluble form of RAGE (sRAGE) [22]. Our results confirm [18,19] that sRAGE was downregulated in COPD patients, stable, and exacerbated (Figure 1C). In particular, levels of sRAGE in stable COPD decreased 2-fold compared to that of controls, while in exacerbated COPD they decreased 2.4-fold compared to that of controls.

Fluorescent AGEs (fAGEs) were increased in stable and exacerbated COPD patients compared to controls and this increase was significantly higher in exacerbated COPD patients compared to that of stable COPD (Figure 1D). In particular, levels of fAGEs in stable COPD increased 2.4-fold compared to that of controls, while in exacerbated COPD they increased 2.5-fold compared to that of controls, and in exacerbated COPD there was a 4% variation in comparison to that of stable COPD.

### 2.2. In Vivo Effects of Carbocysteine on Symptoms and Functional Parameters in Mild Exacerbated COPD Patients

Our results show that miR-21, IL-8, sRAGE, and fAGEs expressions were altered in exacerbated COPD patients. For this reason, we designed a study aimed to assess the effects of carbocysteine in addition to background therapy (LAMA+LABA+ICS) in mild AECOPD patients. We evaluated clinical/functional, inflammatory, and oxidative stress parameters at visit 1 (V1) and at visit 2 (V2) of mild AECOPD patients treated with or without carbocysteine.

**Figure 2:**
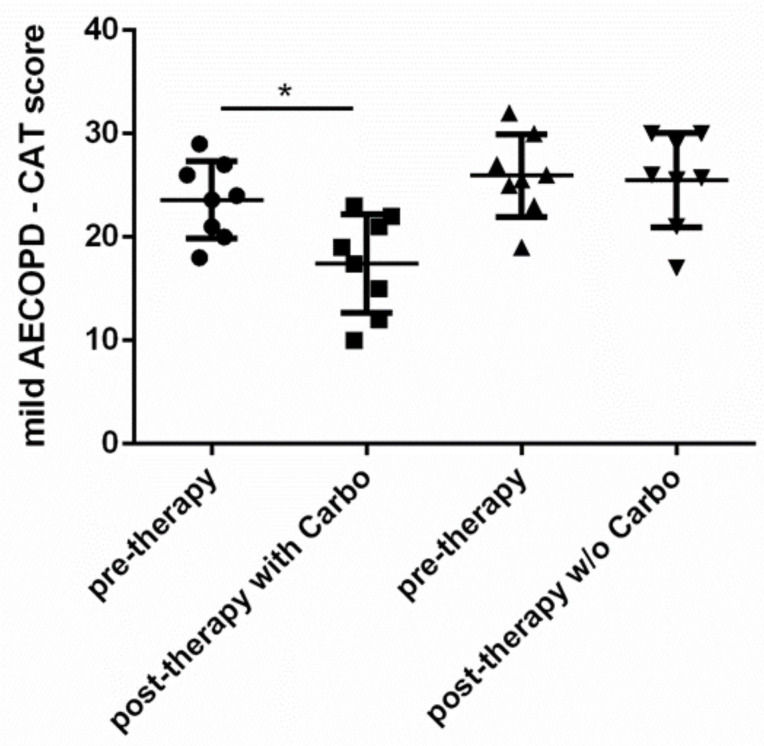
Evaluation of symptoms. Symptoms of mild AECOPD treated with or without (*w/o*) carbocysteine (Carbo) were evaluated by CAT at V1 (pretherapy) and at V2 (post-therapy). Mild AECOPD with Carbo *n* = 8, *w/o* Carbo *n* = 8. * *p* < 0.05 Wilcoxon test.

The results showed that carbocysteine (Carbo) was able to improve CAT symptom score (Figure 2) (26% of variation in comparison to pretherapy).

**Figure 3:**
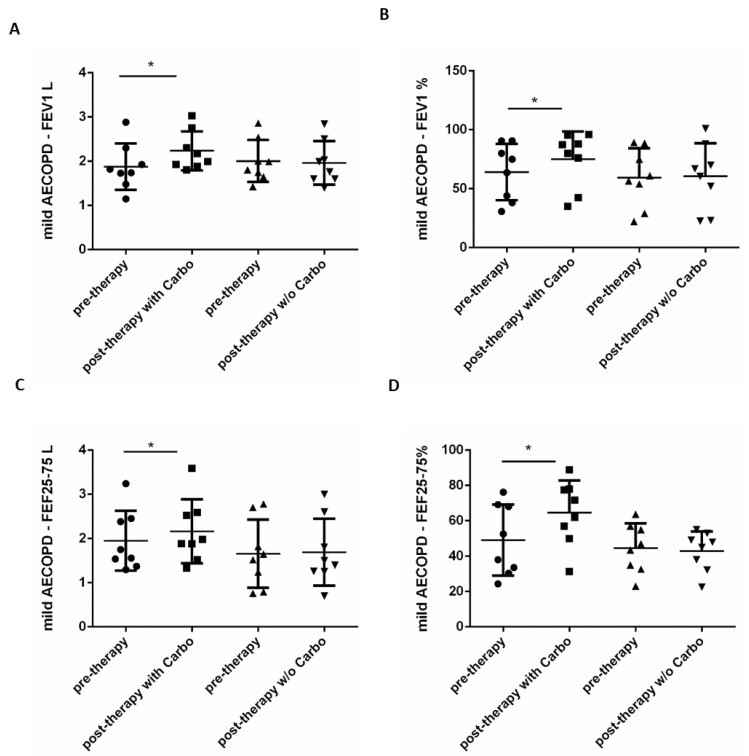
Evaluation of FEV1 and FEF25–75 in mild AECOPD before and post-therapy with or without carbocysteine. FEV1 and FEF25–75, were evaluated in mild AECOPD treated with or without (*w/o*) carbocysteine (Carbo) by spirometry at V1 (pretherapy) and at V2 (post-therapy). FEV1 and FEF25–75 were indicated in liter (FEV1 L) (**A**), (FEF25–75 L) (**C**) and in percentage (FEV1%) (**B**), (FEF25–75%) (**D**). Mild AECOPD with Carbo *n* = 8, *w/o* Carbo *n* = 8. * *p* < 0.05 Wilcoxon test.

With regard to functional parameters, carbocysteine increased both FEV1 (Figure 3 A,B) as well as FEF25–75% (Figure 3C,D). Carbo-treated patients displayed 18% of variation in comparison to pretherapy for FEV1 in liter; 17% of variation in comparison to pretherapy for FEV1 in%; 10% of variation in comparison to pretherapy for FEF25–75 in liter and 31% of variation in comparison to pretherapy for FEF25–75 in %. The group of patients, who did not receive carbocysteine treatment, did not show any variation in the functional parameters before and after therapy (Figure 3A–D).

### 2.3. In Vivo Effects of Carbocysteine on Circulating miR-21 and IL-8 in Mild Exacerbated COPD Patients 

**Figure 4:**
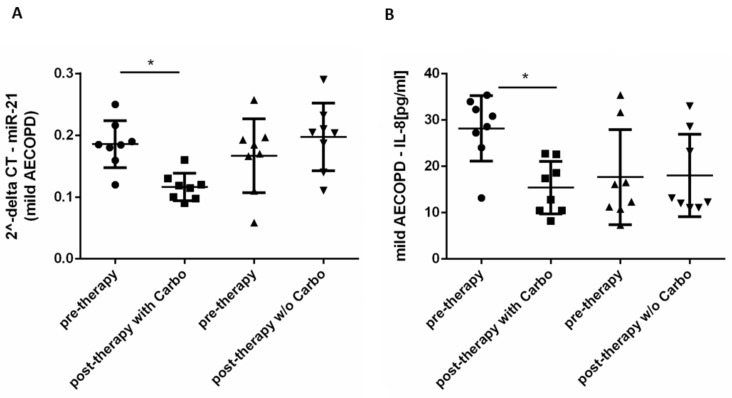
MiR-21 and IL-8 levels in mild AECOPD before and post-therapy with or without carbocysteine. MiR-21 expression and IL-8 levels were evaluated in mild AECOPD treated with or without (*w/o*) carbocysteine (Carbo) at V1 (pretherapy) and at V2 (post-therapy) (**A**) Total RNA was purified from patient’s serum, and miR-21 expression was assessed by real-time PCR. Results are expressed as 2^-deltaCT. (**B**) Measurement of IL-8 in serum was determined by ELISA. Results are expressed as pg/mL. Mild AECOPD with Carbo *n* = 8, *w/o* Carbo *n* = 8. * *p* < 0.05 Mann–Whitney test.

We assessed also circulating miR-21 and IL-8 as inflammatory parameters. After therapy with carbocysteine, mild AECOPD patients showed significantly reduced circulating miR-21 levels (Figure 4A) with a variation of 37% in comparison to pretherapy. Looking at the group of patients who did not receive carbocysteine treatment, the results showed no changes in miR-21 levels (Figure 4A). Interestingly, in mild AECOPD patients that received carbocysteine treatment, before the treatment, miR-21 (2^-deltaCT) inversely and significantly correlated with FEV1 values (see Table 1).

IL-8 levels in the serum of patients treated with carbocysteine were significantly reduced (Figure 4B) (45% of variation in comparison to that of pretherapy). This was not shown in the group that received the therapy without carbocysteine (Figure 4B).

### 2.4. In Vivo Effects of Carbocysteine on Systemic sRAGE and fAGEs Levels in Mild Exacerbated COPD Patients

**Figure 5:**
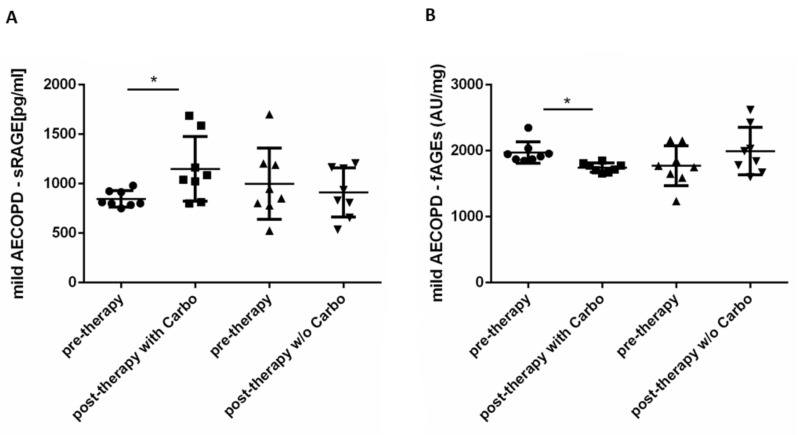
sRAGE and fAGEs expression in mild exacerbated COPD before and post-therapy with or without carbocysteine. sRAGE and fAGE levels were evaluated in mild AECOPD treated with or without (*w/o*) carbocysteine (Carbo) at V1 (pretherapy) and at V2 (post-therapy). (**A**) Measurement of sRAGE in serum was determined by ELISA. Results are expressed as pg/mL. (**B**) Measurement of fAGEs in serum was determined by fluorescence spectroscopy analysis. Results are expressed as Arbitrary Units (AU)/mg of total protein. Mild AECOPD with Carbo *n* = 8, *w/o* Carbo *n* = 8. * *p* < 0.05 Mann–Whitney test.

sRAGE is a decoy receptor that sequestrates RAGE ligands and prevents inflammatory responses [23]. The administration of carbocysteine for 20 days significantly increased circulating sRAGE levels (Figure 5A) with a 35% variation in comparison to that of pretherapy and reduced fAGEs expression (Figure 5B) in mild AECOPD with an 11% variation in comparison to that of pretherapy. Looking at the group of patients who did not receive carbocysteine treatment, the results showed no changes in sRAGE and fAGEs expression (Figure 5A,B).

## 3. Discussion

Frequent AECOPD drive clinical and functional decline in COPD and are associated with accelerated loss of lung function, increased mortality, decreased health-related quality of life and significant economic costs [24]. Recently, clinical research is evaluating the possibility of using drugs that complement traditional therapies to reduce frequency and duration and to manage these episodes of disease exacerbation [2]. Mucolytic agents are considered good candidates for this purpose.

Carbocysteine is one of the most used mucolytic drug with anti-inflammatory and antioxidant action [6,7]. There is limited evidence demonstrating the in vivo anti-inflammatory or antioxidant activities of carbocysteine in COPD or asthmatic patients. Carpagnano G.E. et al. [25] demonstrated that carbocysteine was able to reduce the concentrations of the inflammatory mediators, 8-isoprostane, and IL-6 in exhaled breath condensate of mild acute and mild stable COPD patients. However, there is limited knowledge on the effects of carbocysteine on systemic inflammatory parameters in the mild AECOPD patients.

In this study we choose to add carbocysteine to background inhalation therapy in a selected group of COPD patients with mucus hypersecretion when we experienced a mild exacerbation with worsening symptoms compared to that of the their stable condition. We demonstrated for the first time that the addition of carbocysteine in mild AECOPD patients is able to improve the inflammatory profile, reducing some proinflammatory (miR-21, IL-8, and fAGEs) and increasing some anti-inflammatory (sRAGE) systemic parameters. The positive effects of carbocysteine on molecular parameters were associated with improved symptoms (CAT scores) and respiratory functional parameters (FEV1 and FEF25–75).

These findings add new information to different studies that demonstrated the efficacy of carbocysteine to reduce the exacerbation rate in asthmatics or COPD patients [6,7,26], while not explaining the molecular mechanisms that might underline this clinical efficacy.

In COPD patients, the use of the CAT validated questionnaire is a valuable tool for assessing the health impact of the disease. The CAT is a short, self-administered questionnaire on quality of life useful in patients with a history of exacerbations to identify patients with additional risk of exacerbations. The results obtained through the CAT have a good correlation with other measures of COPD severity such as spirometry airflow measures or pulmonary function. Forced Expiratory Volume in 1 s (FEV1) is a parameter used to evaluate the obstruction of large airways while Forced Expiratory Flow 25–75% (FEF25–75) is a more sensitive parameter than FEV1 in the detection of obstructive small airway disease [27]. Our results demonstrated that the administration of carbocysteine improves CAT symptom scores and increases both these functional parameters leading to an improvement of both proximal and distal airway obstruction. In previous studies, improvement in functional parameters due to the use of mucolytic drugs, including carbocysteine, was not always observed in COPD patients. This is justified by the fact that these studies were not limited to specific COPD phenotypes that may most benefit from the use of mucolytics, such as patients with chronic cough and sputum [28]. Although the present study assessed a small sample size of COPD patients, it has the merit to assess the clinical and molecular effects of carbocysteine in a well-selected COPD patient population with mucus hypersecretion and frequent exacerbations.

In these selected patients, carbocysteine may have the ability to ameliorate both symptoms (CAT score) and functional parameters. Airway obstruction is the result of altered tissue homeostasis due to increased lung inflammation. Within the lungs almost all biological processes including development and homeostasis, viral infection, inflammation, and pulmonary disease are regulated by miRNAs. miRNAs are dysregulated in smoking-associated diseases [29]. Smoking can potentiate inflammatory processes by affecting the expression of miRNAs that play a key role in immune responses. In a mice experimental model, 4 and 24 weeks of smoke exposure increased the levels of several miRNAs in lung tissues, including miR-21 [30]. Previous studies showed that cigarette smoke upregulates miR-21 expression in airway epithelial cells [15] and heavy smokers have higher miR-21 levels than that of healthy controls [31]. For this reason, in the present study, to better understand the impact of mild AECOPD in the studied molecular and clinical events, we selected an exsmoker study population of COPD (stable or exacerbated).

Upregulation of miR-21 is associated to increased IL-8 expression and release [15]. IL-8 is the most important chemokine involved in the recruitment of neutrophils at the airway damage site triggering innate immunity responses [32]. IL-8 could be considered a good biomarker to follow COPD patients during exacerbations because some studies [33] demonstrated that exacerbated patients exhibit higher levels of circulating IL-8 than stable COPD patients. Furthermore, systemic levels of IL-8 are higher in COPD patients hospitalized for severe exacerbations so the assessment of IL-8 in serum could help to predict patients who may experience treatment failure and have a worse lung function [34]. Our results confirmed these findings and showed an increase in IL-8 levels in COPD patients in comparison to that of control group. In addition, our results showed that IL-8 is higher in AECOPD patients compared to stable COPD. The same results were obtained for miR-21 although there was only a trend difference between AECOPD and stable COPD patients. Nevertheless, the literature confirms the correlation between miR-21 and IL-8 [15], and our results showed the correlation of miR-21 with airway obstruction. For all these reasons, we evaluated the effects of carbocysteine also on miR-21.

The most important result herein reported is that carbocysteine therapy is capable of significantly reducing circulating miR-21 and IL-8 levels in a specific phenotype of COPD patients characterized by frequent exacerbations and mucus hyperproduction.

Smoking and oxidative stress promote accelerated formation and accumulation of AGEs, causing inflammation and local tissue damage either directly or by binding the RAGE [35]. RAGE knockdown decreased NF-κB and NLRP3 activation, leading to reduced IL-1β and oxidant production, while it increases thiol pools [36]. Emerging evidence suggests that RAGE signaling is involved in the pathophysiology of COPD. COPD patients showed lower plasma sRAGE levels and higher plasma AGEs levels compared to that of controls, and these values are associated with decreased lung function [35]. Neutrophilic airway inflammation in asthma and COPD is associated with reduced sRAGE [37], and this is in turn associated with COPD disease severity [38]. Herein, we demonstrated that the administration of carbocysteine in mild AECOPD patients is able to increase the circulating sRAGE and to reduce the circulating fAGEs.

Several in vitro studies support the combined use of carbocysteine with steroids to better control inflammation and oxidative stress. In particular, it was demonstrated that carbocysteine counteracts some mechanisms related to steroids resistance induced by cigarette smoking and typical of severe COPD [39,40]. Similar molecular mechanisms could explain the results obtained in this in vivo study, but further studies are needed to investigate them and extend biological and physiological assessments to a larger group of patients. However, the COVID-19 pandemic limited the possibility of extending this study. There are further limitations regarding this pilot study, and these deserve further dedicated studies. In detail, the following should be assessed: (a) the effects of carbocysteine in reducing AE (frequency/severity/length); (b) the effects of carbocysteine in COPD patients with different disease severity when stable or in patients with moderate or severe AECOPD; (c) the impact of smoking on carbocysteine effects; (d) the inflammatory parameters in airway samples; and (e) the impact of comorbidities.

## 4. Materials and Methods

### 4.1. Recruited Study Population

Forty-two subjects were recruited at IRIB-CNR Clinic. The study was approved by the reference Policlinico “Paolo Giaccone” Ethic Committee (n.10/2017-11/15/2017) in accordance with the Declaration of Helsinki. Informed written consent was obtained from each subject.

The subjects were grouped in controls: stable COPD and exacerbated COPD, as shown in Table 2. All subjects had negative skin tests for common aeroallergen extracts and had no history of asthma and/or allergic rhinitis. No significant differences were found for cigarette pack/year, FEV1%, FEF25–75%, BMI, between COPD groups and between the groups receiving or not carbocysteine.

Healthy, nonsmoker subjects were recruited as controls (*n* = 9). COPD patients were classified based on GOLD Guidelines 2019 (https://goldcopd.org/pocketguidereferences/gold-2019-pocket-guide-references/, accessed on 7 February 2022): FEV_1_ less than 80% of reference, FEV_1_/FVC less than 70%, and bronchodilatation effect less than 12%. Stable COPD patients were GOLD 2 (Stable COPD) and AECOPD patients were GOLD 2–3 (Exacerbated COPD) stage. All COPD patients (stable and exacerbated) are exsmokers who quit smoking at least two years prior to the commencement of the study (smoking history as pack/years were reported in Table 2). Stable COPD patients (*n* = 9) were treated with bronchodilators, long-acting muscarinic antagonists (LAMA), and long-acting beta-adrenergic agonists (LABA). Stable COPD patients did not manifest mucus hypersecretion, and therefore did not require carbocysteine therapy [5].

Exacerbated COPD patients were all characterized by mucus hypersecretion and experienced frequent exacerbations (2–3 per year) and were treated with LAMA and LABA combined with inhaled corticosteroids (ICS). Patients kept a symptom diary which included these questions: (1) is your sputum difficult to cough up?; (2) has the amount or color of sputum changed?; (3) is your cough more troublesome?; and (4) have you noticed the increase in any other COPD-related symptoms? The patients were instructed to alert their pulmonologist to report symptom variation, and when they responded yes to at least two questions the visit was programmed (day 1). During the visit 1 (V1) (day 2), patients were classified according to Anthonisen criteria and recruited for the study. Recruited exacerbated COPD patients (*n* = 24) included both mild (*n* = 16) and moderate (*n* = 8) exacerbated COPD. Only mild exacerbated COPD were assessed for evaluating carbocysteine effects (*n* = 16). Eight moderate exacerbated COPD requiring systemic corticosteroids and/or antibiotic therapy were excluded from the study, because these drugs might act as confounding variables that did not allow to discriminate the effects of carbocysteine.

### 4.2. Study Design

Open prospective observational real-life study aimed to demonstrate in vivo antioxidant and anti-inflammatory effects of carbocysteine for the management of mild AECOPD.

Mild exacerbated COPD, randomly chosen (1:1), received (*n* = 8) or not (*n* = 8) therapy with a single daily dose of carbocysteine lysine salt (2.7 g/day) (Dompè, Milan, Italy) for 20 days in addition to background therapy (LAMA+LABA+ICS). The “20 days” interval was selected for two main aspects: (a) it is a time interval that could be sufficient to evaluate the efficacy of carbocysteine as a drug for the management of mild AECOPD; (b) to assess patients at the end of an exacerbation that was reported to last two weeks [41].

**Figure 6:**
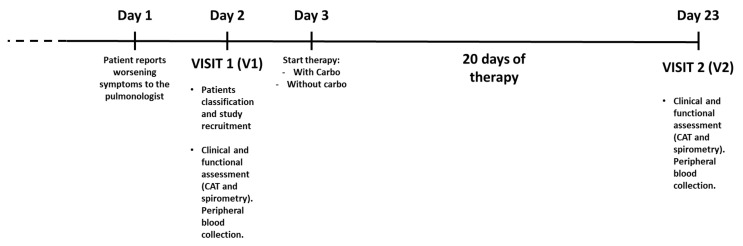
Scheme of study design.

The carbocysteine was added to background therapy on the third day (day 3) of worsening of symptoms in the carbocysteine-treated group. Mild AECOPD patients were evaluated at visit 1 (V1) and at visit 2 (V2) for clinical and functional assessment (COPD Assessment Test (CAT) questionnaire and spirometry) and for peripheral blood evaluations (miR-21, IL-8, sRAGE, fAGEs). The scheme of the study design is reported in Figure 6. For all the COPD recruited patients clinical and functional assessment as well as blood collection were performed before daily inhalation therapy.

### 4.3. Evaluation of Clinical and Functional Parameters

Mild AECOPD patients were evaluated at V1 and V2 for symptoms and functional parameters. The functional parameters of mild AECOPD patients during stable period were: FEV1%: 59.05 ± 11.98, FEF25–75%: 62.83 ± 13.09. Patient symptoms were collected using the COPD Assessment Test (CAT) questionnaire. The functional parameters, Forced Expiratory Volume in 1 s (FEV1) and Forced Expiratory Flow (FEF25–75), were evaluated by microQuark spirometry (Cosmed s.r.l. Albano Laziale, Roma, Italy). Spirometry was performed in accordance with the American Thoracic Society/European Respiratory Society (ATS/ERS) guidelines [42].

### 4.4. Evaluation of Serum miR-21

Total RNA was purified from human serum using miRNeasy Serum/Plasma kit (Qiagen, Hilden, DE) following the manufacturer’s instruction. As spike-in, we used cel-miR39-3p and we added 1nM of its mimic sequence (mirVana™ miRNA Mimic, ID: MC10956, Applied Biosystems, Foster City, CA, USA) for each sample.

For reverse transcription of miR-21-5p and cel-miR39-3p, RNA was reverse transcribed using specific primers (Taq-Man™ MicroRNA Assay, miR-21-5p Assay ID: 000397 and cel-miR-39 Assay ID: 000200, Applied Biosystems, Foster City, CA, USA) and the TaqMan miRNA RT-Kit (Applied Biosystems, Foster City, CA, USA) following the manufacturer’s instruction. qRT-PCR of miR-21-5p and cel-miR39-3p transcripts was carried out on Step One Plus Real-time PCR System (Applied Biosystems, Foster City, CA, USA) using specific TaqMan MicroRNA Assays (Applied Biosystems, Foster City, CA, USA). The results were expressed as 2^-delta CT. We obtained the delta CT values subtracting the CT values of cel-miR39-3p from the CT values of the miR-21-5p.

### 4.5. Measurement of IL-8 and sRAGE

The measurement of IL-8 [15] and sRAGE [18] in serum were determined using enzyme-linked immunosorbent assays (ELISA) (R&D Systems, Minneapolis, MN, USA) according to the manufacturer’s instructions. The data were expressed as pg/mL.

### 4.6. Measurement of Fluorescent AGEs

Quantitative fluorescence spectroscopy analysis was used to measure fAGEs concentration in serum [43,44]. Sera were centrifuged at 14,000 g for 1 h and then diluted 50-fold with phosphate-buffered saline (PBS). The fluorescence intensity was recorded at an emission wavelength of 440 nm after an excitation at 350 nm using GloMax® Discover Microplate Reader (Promega Corporation, Madison, Wisconsin, USA). PBS solution was used as blank. The data were expressed in arbitrary units (AU) and normalized to the total protein amount (AU/mg). The total protein was measured by the Bradford assay. Each sample was analyzed in triplicate.

### 4.7. Statistics

Data were expressed as mean ± SD. Mann–Whitney test and Wilcoxon test were used. A *p*-value minor than 0.05 was considered being statistically significant.

## 5. Conclusions

The present study provides further evidence to support the efficacy of carbocysteine in the management of mild AECOPD patients. We propose that this action is in part due to the effects of carbocysteine on some systemic molecular events related to inflammation and oxidative stress with a relevant role in the progression of COPD and that are not properly controlled by basic inhalation therapy.

## Figures and Tables

**Table 1:** Correlation between miR-21 and FEV1 values in mild AECOPD post-treatment with carbocysteine.

	Correlation Coefficient miR-21 (2^-deltaCT)	*p* Values
FEV1(l)	−0.7769406	0.031
FEV1(%)	−0.8189638	0.019
FEF 25–75(l)	−0.6835159	0.078
FEF 25–75(%)	−0.718366	0.055

**Table 2:** Patient demographics.

	Controls	Stable COPD	Exacerbated COPD
*N*	9	9	24
Age	50 ± 10	62 ± 8	58 ± 18
Gender (M/F)	4/5	5/4	12/12
pack/year	-	35 ± 10.31	40.7 ± 11.86
FEV1(%predicted)	106.22 ± 12.21	57.50 ± 11.03	48.31 ± 16.77
FEF25–75(%predicted)	101.78 ± 12.34	61.73 ± 10.43	50.24 ± 17.09
GOLD		2	2–3
BMI	27.81 ± 3.11	28.05 ± 2.26	28.51 ± 6.92

## Data Availability

The data used to support the findings of this study are included within article.

## Abbreviations

COPD	chronic obstructive pulmonary disease
AECOPD	acute exacerbation chronic obstructive pulmonary disease
Carbo	carbocysteine
miRNAs	microRNAs
CSE	cigarette smoke extract
fAGEs	fluorescent advanced glycation end products
sRAGE	soluble receptor AGE
CAT	COPD Assessment Test
FEV1	Forced Expiratory Volume in 1 s
FEF25–75	Forced Expiratory Flow
